# Plastic pollution of four understudied marine ecosystems: a review of mangroves, seagrass meadows, the Arctic Ocean and the deep seafloor

**DOI:** 10.1042/ETLS20220017

**Published:** 2022-10-10

**Authors:** Bruno Andreas Walther, Melanie Bergmann

**Affiliations:** Alfred Wegener Institute Helmholtz Centre for Polar and Marine Research, Bremerhaven, Germany

**Keywords:** ecological impact, ecosystems, plastic pollution

## Abstract

Plastic pollution is now a worldwide phenomenon affecting all marine ecosystems, but some ecosystems and regions remain understudied. Here, we review the presence and impacts of macroplastics and microplastics for four such ecosystems: mangroves, seagrass meadows, the Arctic Ocean and the deep seafloor. Plastic production has grown steadily, and thus the impact on species and ecosystems has increased, too. The accumulated evidence also indicates that plastic pollution is an additional and increasing stressor to these already ecosystems and many of the species living in them. However, laboratory or field studies, which provide strong correlational or experimental evidence of ecological harm due to plastic pollution remain scarce or absent for these ecosystems. Based on these findings, we give some research recommendations for the future.

## Introduction

Plastic pollution has become a hallmark of the Anthropocene because of three features: it is an entirely man-made product, the pollution is growing exponentially because increasing production is not coupled with sufficient waste management and recycling, and the pollution is essentially irreversible [1,2]. This irreversible plastic pollution has begun to impact populations, species and ecosystems to varying degrees, running the entire spectrum from no harm to severe harm [3–7]. Persson et al. [8] recently suggested that we have already crossed the planetary boundary for plastics together with other chemical entities.

Since Thompson et al.’s [9] landmark publication, research on plastic pollution has also been growing exponentially [6]. However, anybody who has reviewed the scientific literature on plastic pollution will quickly realise that most research focuses on the effects of plastic pollution alone, without the presence of other stressors. Moreover, most research only focuses on one aspect of plastic pollution, e.g. only on the effects of macroplastics, or microplastics, or nanoplastics. On top of that, much research is mainly restricted to the level of organisms or below (e.g. organs, tissues, or cells) and conducted in laboratory and mesocosm experiments [10]. This is not a critique: As research on plastic pollution is a relatively recent and still emerging field, it is only natural to focus on understanding the isolated effects of plastic pollution first.

However, in the real world, plastic pollution is not acting alone, but it just adds another impact to the already existing cocktail of man-made impacts on marine ecosystems, such as global heating, ocean acidification, eutrophication, deoxygenation, overharvesting, shipping and underwater noise, habitat destruction and fragmentation, and chemical pollution [6,11–16]. These multiple stressors acting together may push already threatened and vulnerable marine ecosystems over the brink with possibly detrimental effects for ecosystem functioning and services [17–19], and we see more and more examples of ecosystems unravelling, collapsing into a much simpler and much less productive state [20–23].

In this review, we focus on three understudied marine ecosystems which are already declining and threatened due to various other man-made impacts and which increasingly bear the additional burden of plastic pollution (see also Supplementary Text S1): mangroves, seagrass meadows and the Arctic. This selection was based (1) on our expertise and (2) the fact that these ecosystems have received less attention than, e.g. coral reefs, which have been the subject of hundreds of publications about plastic pollution and its effects (reviewed in [6,10,24–28]). We further included the deep sea floor, which may not be threatened yet, but where research points towards increasing disturbances due to man-made impacts, and which due to logistical reasons also remains understudied.

## Mangroves

Mangroves provide many important ecosystem services to coastal communities, but are nevertheless disappearing because of a multitude of man-made threats (Supplementary Text S2). Another threat has recently arisen with plastic pollution [29,30].

### Macroplastics

**Figure 1.**
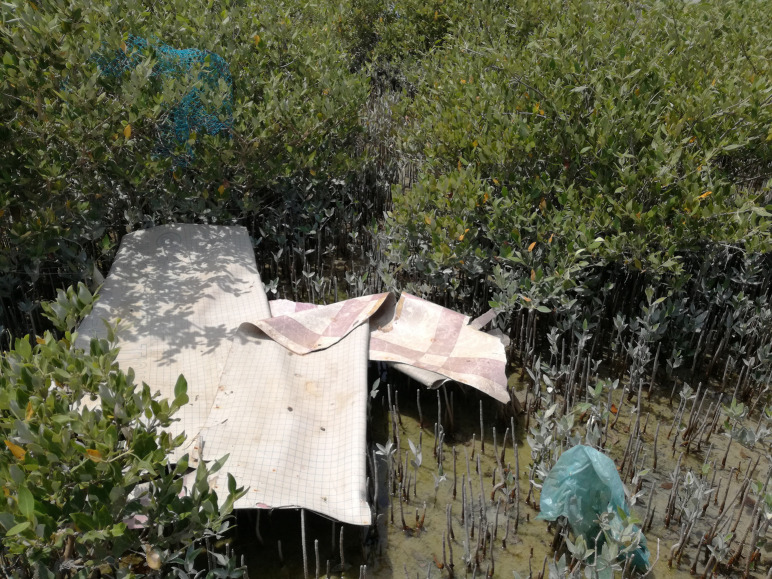
Macroplastics smothering mangroves. Several large macroplastic items smothering the roots and branches of a Red Sea mangrove forests [33] (photo credit: Cecilia Martin).

**Figure 2.**
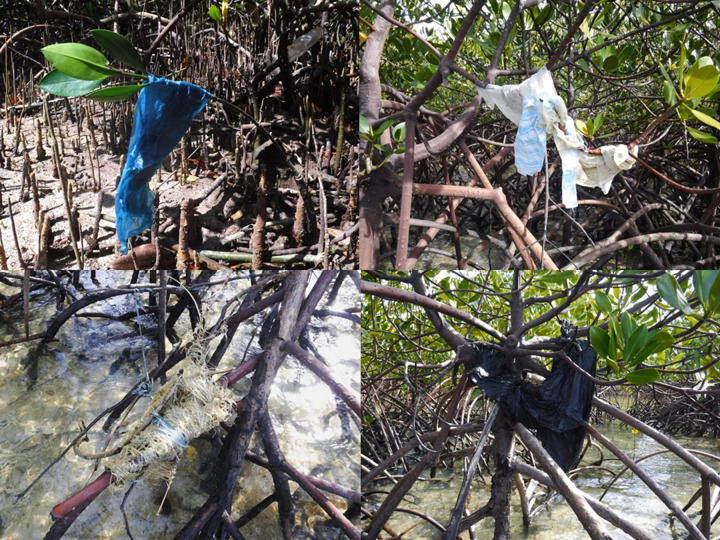
Macroplastics caught in mangroves. Four examples of macroplastic items caught in the roots and branches of Philippine mangrove forests (location: Mati, Davao Oriental, Mindanao; date: December 2017) (photo credit: Neil Angelo S. Abreo).

Mangroves seem to be especially impacted by plastic pollution because their complex aerial root systems give mangrove forests a high structural complexity which, in turn, creates a high trapping potential for marine debris [29,31] most of which is made up by plastics [32–35] (Figures 1, 2). The enhanced trapping ability was supported by the observation that debris density was positively related to tree density ([33,36] but see [37–39]) and that mangroves and tidal marshes had higher plastic abundances than tidal flats and seagrass meadows [30]. Another reason for these high pollution levels is that 54% of mangrove habitats are within 20 km of a river mouth, many of which belong to the most polluted in the world, and much of this plastic pollution remains — at least initially — close to the river mouth and adjacent coastline [40,41]. Consequently, some of the highest densities of plastic pollution ever reported come from mangroves [32,35,39,42].

However, very few studies have so far related debris density to the mangroves’ condition. In Indonesian mangroves, four indicators of tree health were negatively correlated with debris density [39]. Similarly, three indices of mangrove health decreased with increasing amounts of debris, although only non-significantly, with variation explained ranging from 6% to 13% [43]. Observations further suggest that plastic debris hindered photosynthesis, smothered and thus suffocated pneumatophores, led to root deformation, disrupted aeration and water movement, which can lead to decreased soil quality, and directly sacrificed mangrove trees, particularly seedlings, because plastics physically broke down seedlings [39,44–51]. What's more, the rehabilitation of mangrove forests can fail due to tree seedlings being smothered by marine debris [35], and seedling survival was negatively impacted by entanglement with fishing lines and plastic shopping bags [52].

The most conclusive study was conducted in mangrove forests along the Javan coast [42]. Plastic debris covered up to 50% of the forest floor at several locations, consequently starving the trees of oxygen. In another Javan locality, marine debris even covered ∼80% of the forest floor (R. Ivonie, in litt. 2022). Van Bijsterveldt et al. [42] also conducted a field experiment which varied the percentage of pneumatophores covered with plastic. Within the six-week period, mangrove trees already displayed significant leaf loss and increased mortality with increasing plastic pollution coverage (0%, 50%, and 100%). The effects of smothering with plastics were thus similar to those of smothering with sediments which can cause negative effects from reduced vigour to death [53].

So far, few effects on animal inhabitants have been documented. For example, several negative effects on the biota in an Indian mangrove forest were reported, that included the sound of plastic bags which during stronger winds scared away water birds [54]. An increase in the percentage of surface covered by garbage inside a mangrove forest was significantly correlated with a decrease in active crab burrows [47]. Crabs were also observed to bury plastic [31], and various mangrove species used debris as a novel habitat [34].

### Microplastics

The growing presence of microplastics in mangroves has been documented in several recent comprehensive reviews [27,30,55–58]. A range of 0–11 256 plastic items/kg (median = 209) was documented in mangrove sediments ([30]; see also [57,58]), and concentrations in mangrove surface water also varied greatly ([57]; Supplementary Text S3). Mangrove sediments have thus acted as a plastic sink with rising concentrations mirroring the exponential increase in plastic production [59].

A positive relationship between the density of mangrove pneumatophores and plastic fibre abundance suggested that the mangroves’ structural complexity may also trap microplastics [60]. This trapping potential was also suggested by the observation that between 11% to 56% of microplastics flowing down a river were intercepted by the mangrove forests bordering the river [61]. A meta-analysis of published experiments demonstrated a strong and negative effect of microplastics on biota abundance and a weak negative effect on mangrove survival [30].

Not surprisingly, microplastics were found in clams, crabs, fishes, molluscs, gastropods, and sponges living in mangroves, with 7% to 100% of the investigated individuals containing microplastics ([30,57,58,62]; Supplementary Text S3).

## Seagrass meadows

Seagrass meadows also provide many important ecosystem services to coastal communities, but are threatened because of a multitude of man-made threats (Supplementary Text S2). Another threat has recently arisen with plastic pollution [63].

### Macroplastics

While the structural complexity of seagrass meadows is obviously less than that of mangroves, they nevertheless also act as a trap for macroplastics. In Mediterranean seagrass meadows, the main area for litter accumulation was the landside edge along the meadows, which demonstrates their trapping potential for macrodebris [64]. Similarly, a few macroplastics were found in Portuguese seagrass habitats, whereas adjacent unvegetated habitats contained none [65]. Across studies, macroplastic densities in seagrass meadows ranged from 0–13.3 items/100 m^2^ [64–66]. Plastic pollution slowed the nitrogen liberation from seagrass detritus with possible effects on nutrient cycling and coastal biogeochemistry [67]. In special circumstances, these seagrass sinks can become a source of macroplastics and microplastics on beaches [68].

Three experimental studies explored the effects of plastics. In a mesocosm experiment, the effects of biodegradable plastic bags on a common Mediterranean seagrass species were examined [69]. After six months, the bags retained 85% of their initial mass and influenced sediment geochemistry and plant growth in complex ways. In another mesocosm experiment, the same seagrass species was exposed to both macroplastics and sedimentation [70]. After 18 months, the macroplastics were still present in the sediments, and they could make seagrasses vulnerable to sedimentation and could reduce plant cover. In another mesocosm experiment, plastic pollution reduced the decomposition rate of eelgrass by 36% and slowed nitrogen liberation from the seagrass detritus [67].

Threatened species may be impacted; e.g. a Philippine seagrass area, which is an important feeding habitat for threatened dugongs was polluted with macroplastics [66].

### Microplastics

**Figure 3.**
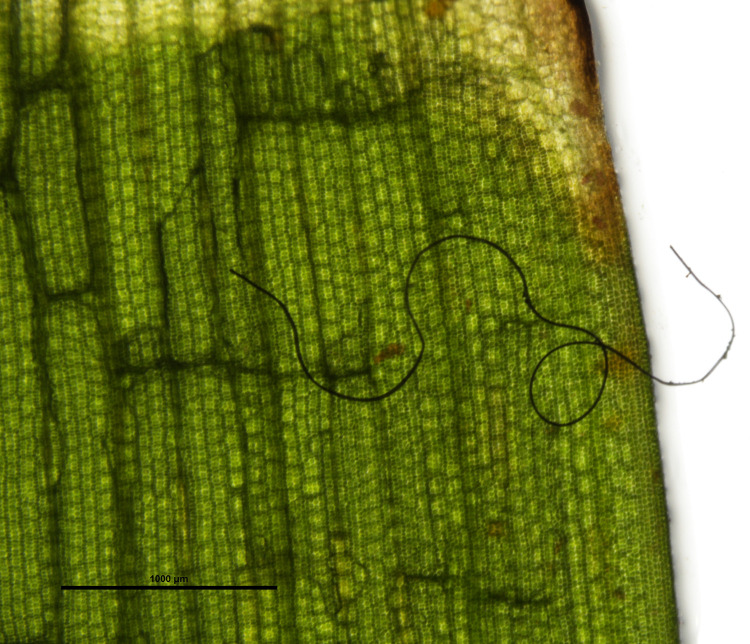
Microplastics caught in seagrasses. A plastic microfiber stuck on a blade of seagrass *Thalassia hemprichii* (photo credit: Nicholas Seng Ren Yang, taken in laboratory in 2020). Their presence suggests another pathway for microplastics to enter into the marine food web, namely, through herbivory.

The growing presence of microplastics in seagrass meadows has been documented in three recent comprehensive reviews [30,63,71]. When compared with mangroves, saltmarshes, and tidal flats, seagrass meadows had the lowest plastic abundance (range 0–1466 items/kg) in sediments from around the world ([30]; Supplementary Text S4). However, when seagrass meadows were compared with adjacent unvegetated plots, sediments of seagrass meadows usually had higher microplastic concentrations, which suggests that seagrass meadows can also act as a sink for microplastics (Supplementary Text S4). The trapping potential of seagrass meadows was also demonstrated by three experimental studies (Supplementary Text S4). Microplastics and leached chemicals were also detected on seagrass blades, the surrounding water, benthos, invertebrates and fishes living in seagrass meadows (Figure 3; Supplementary Text S4).

## Arctic

Polar regions are the cold stores of the planet that stabilise the Earth's climate. However, they are particularly threatened by climate change. Arctic temperatures have increased four times faster than the global average [72], causing a massive decline of sea ice. In addition to ecological impacts, this decline has boosted anthropogenic activities, such as fisheries and shipping, exerting further pressure on Arctic ecosystems [73–76] in terms of harvesting, noise and chemical pollution [77,78].

**Figure 4.**
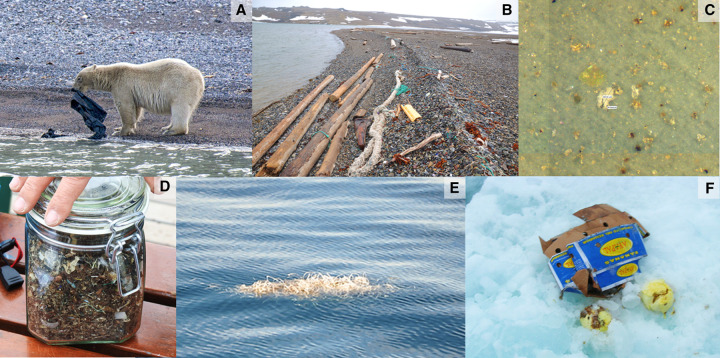
Anthropogenic debris in the Arctic. Anthropogenic debris observed on Svalbard, Norway. (**A**) Polar bear inspecting a plastic bin liner on Lomfjorden (photo credit: Andreas Alexander). (**B**) Plastic debris beached at Sørvika (photo credit: Birgit Lutz). (**C**) Microplastic particle (polypropylen) detected in Arctic snow sample (photo credit: Sophia Mützel). (**D**) Sediment sample of Lomfjorden with many mesoplastic and microplastic fragments (photo credit: Birgit Lutz). (**E**) A bundle of plastic straps floating at the sea surface (photo credit: Birgit Lutz). (**F**) Cardboard box for bananas and food waste dumped onto an ice floe in the Fram Strait (photo credit: Melanie Bergmann, AWI).

Despite its remoteness, plastic pollution has become pervasive in Arctic ecosystems from the sea surface to the water column, seafloor, beaches, cryosphere, lakes, and rivers [79] (Figure 4) since the first records in 1957 [80]. Several studies highlighted that plastic pollution is particularly abundant in the Arctic [81–84], which supports projections of a Nordic accumulation area [85]. Indeed, time-series data spanning 13 years showed that plastic debris increased more than seven-fold between 2004 and 2017 on the Arctic seafloor [86]

### Macroplastics

Like elsewhere, large debris entangles Arctic biota, e.g. several seabird species on Svalbard and in the Russian Arctic, where it is also incorporated into nests [87,88]. Terrestrial wildlife (Arctic fox, polar bear, reindeer), marine mammals (bowhead whale, bearded and harbour seal), fish (Atlantic cod, Greenland halibut) and snow crabs also suffered entanglements [87,89–91]. On the deep seafloor, plastic entangled up to 31% of the sponge colonies of *Caulophacus arcticus* [86], which could interfere with feeding and oxygen uptake. Plastic debris also carries rafting organisms to Arctic ecosystems, whose communities are already in transition due to rapid heating [92,93]. If rafting species establish themselves, this in turn would affect Arctic biodiversity. The re-appearance of the blue mussel in Svalbard waters was attributed to rafting on floating debris [93]. Furthermore, current records show that 31 Arctic species, including very long-lived hooded seals and Greenland sharks (classified as Vulnerable), ingest plastic debris [79], which could weaken them.

### Microplastics

Microplastic can be ingested by a wider range of organisms and were thus reported from zooplankton species at the base of the food web [94], benthic invertebrates [95,96], fishes [97], seabirds [98], up to walruses, Belugas, and other whales [97,99]. Ingested microplastics can leach chemicals and weaken organisms, which cannot gain energy from ingested plastics, especially when they cannot be excreted [100]. While the effects on Arctic ecosystems are poorly understood, it is conceivable that they are similar to those reported from biota found elsewhere [79]. It could even be argued that effects exert a greater pressure on organisms, which already suffer from rapid environmental change. For example, timing mismatches between ice algal and pelagic blooms and unfavourable thermal conditions can decrease zooplankton abundance [101]. Starving zooplankton organisms that ingest microplastic instead of phytoplankton are likely weakened further, especially at critical early-life stages [94], which could reverberate throughout the food chain. Species that are closely associated with the ice edge, such as certain amphipods and polar cod [94,102], could be particularly prone to impacts as the sea ice stores extremely high quantities of microplastics [82] that are released to the underlying water and ice algae (M. Bergmann, unpublished data) during the melting period. Similarly, deposit-feeding organisms living underneath the marginal ice zone process sediments that can contain up to 13 000 microplastics per kg sediment [83]; thus, these organisms very likely ingest significant quantities of microplastics although this has yet to be confirmed.

Several Arctic species that interact with plastic debris are listed as vulnerable (e.g. Atlantic cod, Greenland shark, hooded seal, polar bear, reindeer, sperm whale, walrus, Leach's storm-petrel, black-legged and red-legged kittiwakes) or near-threatened (Cassin's auklet, common eider, sooty shearwater) by the IUCN and could thus be at particular risk. Furthermore, risks are likely underestimated because many species and ecosystems remain poorly studied in this remote and challenging part of the world.

Another emerging topic of concern is the effect of microplastics on climate change processes and biogeochemical cycles, which urgently requires further research [79].

## Deep seafloor

The deep seafloor is Earth's largest habitat by area (∼50%) and supports very diverse ecosystems [103], which includes the long-term storage of carbon fixed in the upper ocean [104]. Although much of it remains unexplored due to technological challenges, research indicates that plastic pollution could be an important stressor [105], which exacerbates the impact of climate change, fishing, mining, hydrocarbon industry, invasive species, and noise pollution [16,106,107]. The first record of plastic debris on the seafloor dates back to 1971 for an Antarctic sponge garden [108].

### Macroplastics

**Figure 5.**
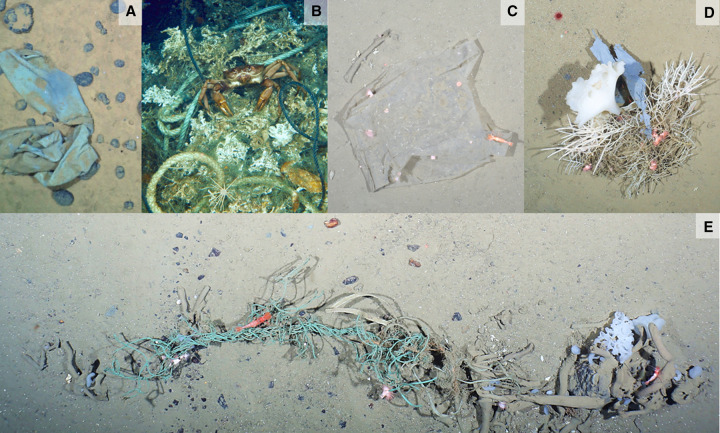
Anthropogenic debris observed on the deep seafloor. Anthropogenic debris observed on the deep seafloor. (**A**) Textile lying on sediments and manganese nodules of the North Pacific seafloor (5900 m water depth). (**B**) Fisheries ropes entangled in cold-water corals off the Lofoten (photo credit: Victor 6000/IFREMER). (**C**) Plastic bag colonised by sea anemones. (**D**) Plastic sheet entangled in sponges *Caulophacus arcticus* and *Cladorhiza gelida*. (**E**) A bundle of plastic ropes with resting *Bythocaris* shrimp on the seafloor of Fram Strait, Arctic (ca. 2500 m water depth) (photo credits except B: Melanie Bergmann, AWI).

The deep seafloor is considered a sink for marine debris [6,109,110] (Figure 5) because about half of the plastic from municipal solid waste exceeds the density of seawater and thus sinks to the seafloor [111]. With time, most of the remainder descends, too, due to degradation, hydrographic, and ballasting processes [112] unless it is stranded. On the deep seafloor, degradation is particularly slow due to low temperatures and the absence of sunlight [113]. Debris is thus observed in most seafloor surveys, including the Mariana Trench [114]; thus it has likely become ubiquitous [105]. Particularly high quantities are found in depressions, such as the Xisha Trough in the South China Sea [115] and canyons [116] as well as in the Mediterranean Sea and the Arctic Ocean [86,117], where quantities have increased five- to seven-fold over time [86,118].

While our knowledge of the impacts of benthic plastic debris is still scarce, Canals et al. [105] suggested that the most distinct impacts on seafloor communities are the coverage of soft sediments, the coverage and entanglement of sessile animals, and the introduction of artificial substrata. Plastic objects can be used for the attachment of encrusting and sessile organisms in otherwise homogeneous muddy environments with few natural hard substrata [74,119,120]. Overall, these introduced objects can locally increase the number of species, but at the same time decrease the numbers and functions of species adapted to this particular environment.

Since drifting plastic debris is often intercepted by sessile suspension feeders, such as sponges or cold-water corals, such marine animal forests were even proposed to become sentinels for monitoring plastic pollution [121]. Indeed, almost up to a third of the sponges from the Arctic seafloor were entangled with litter, and entanglement and debris quantities increased over time on the seafloor [86]. Such entanglements could inflict injury, disease, starvation, and death, as observed for Antarctic sponges [108] and cold-water corals [122]. In addition, derelict fishing gear can continue to catch organisms, as did an old fishing net, which had caught crabs on the deep Mediterranean seafloor [123].

Soft-sediments cover vast areas of the seafloor and are inhabited by diverse infaunal organisms. Plastic lying upon sediments can affect geochemical processes at the sediment-water interface and biota. Experiments in coastal settings produced oxygen-deprived conditions underneath plastic bags and a reduced availability of food which resulted in lower densities and diversities of sediment-inhabiting invertebrates [69,124].

### Microplastics

Microplastic pollution has infiltrated the deep seafloor globally [125]. Smaller-sized particles sink to the seafloor more rapidly because their higher surface-to-volume ratio increases fouling, which adds weight and thus accelerates sinking [126], especially when incorporated into aggregates and faeces [112]. Microplastic levels in deep sea sediments are in a similar range or higher than in shallower areas. Again, depressions, such as canyons and trenches, harbour the highest levels [125], and currents can transport particles to accumulation areas that coincide with biodiversity hotspots [127]. Woodall et al. [128] described for the first time how deep sea sediments have become an important sink for microplastics. A recent meta-analysis confirmed the overall result but found the highest concentrations at 200–2000 m depths instead of in depressions [129]. Stratigraphic analyses of sediments from different regions demonstrated pollution levels increasing over time [130–132].

Microplastics were recorded in fish and invertebrates from the Mariana Trench [133], mid-Atlantic and SW Indian Ocean [134], north and western Pacific [135,136], South China Sea [137], NE and S Atlantic [138,139] and the NW Atlantic [140], including museum specimens from 1975 [141]. In the Rockall Trough, 48% of the invertebrates examined contained microplastics, highlighting their widespread prevalence [142]. The frequent ingestion of microplastics and associated chemicals instead of food could lower an animal's energy reserves [100], which could be critical for animals of already food-limited environments, such as the deep sea. Exposure to microplastics affected calcification rates of cold-water corals [122], which are keystones in terms of structuring benthic habitats. However, since it is almost impossible to conduct mesocosm or laboratory experiments on deep sea animals, the effects remain largely in the dark. In addition, next to nothing is known about the effect of microplastics on the biogeochemistry of sediments including carbon sequestration [143], although microplastics in shallow saltmarsh sediments altered microbial community composition and nitrogen cycling processes [144]. Birarda et al. [145] suggested that plastics and their chemicals could also alter bio-mineralization processes in benthic foraminifera, an important ecosystem function. Given the high microplastic concentrations recorded in certain regions [83,146–148], this could have serious effects on important ecosystem services because quantities already substantially exceed the assumed safe concentration of 540 particles/kg sediment, e.g. by up to 25-fold in the Arctic deep sea [149].

The available evidence indicates that plastic pollution has become pervasive in the deep sea, which thus constitutes an important sink with Martin et al. [129] estimating that 25–900 million metric tons of nonfibrous microplastics and mesoplastics accumulated globally in marine sediments from 1950 to 2010. Given the scarcity of effect data it is difficult to gauge the ecological impact. However, biota inhabiting hot spots with high pollution levels could already suffer impacts as could sensitive species such as deep sea corals and sponges.

## Future research recommendations

Together with other threats, plastic pollution undoubtedly harms marine species, biodiversity and ecosystems [4,6,150]. Above, we summarised what is known about four marine ecosystems which remain understudied. In all four ecosystems, an increasing presence of plastic pollution, both as macroplastics and microplastics, has been reported. The available evidence also suggests that plastic pollution can be considered an additional and increasing stressor to already stressed ecosystems [6,25,151]. However, still too little is known about the ecological impacts of plastic pollution. Therefore, we provide a few research recommendations below which we feel are of particular importance, based on the results from our review and our best professional opinion.

### Brief summary of ecological harm

For mangroves, we have a number of observational studies which reported harm. However, to the best of our knowledge, we have so far only three correlational studies [39,43,47] and one experimental study [42] for the impacts of macroplastics and one meta-analysis for the impacts of microplastics [30]. For seagrass meadows, there were only three mesocosm experiments [67,69,70] for the impacts of macroplastics and none for microplastics.

For the Arctic and the deep seafloor, the documented impacts of plastic pollution are the usual suspects: coverage, entanglement, ingestion, and rafting of potentially invasive species (these general impacts apply, of course, to most other marine ecosystems, too, such as coral reefs, mangroves, seagrasses, etc.) [6]. However, while these impacts are real, there is no correlational or experimental evidence of harm caused to populations or ecosystems of the Arctic or the deep seafloor so far.

### Establishing ecological harm

This meagreness of evidence entails an urgent research need to establish such harm in correlational and experimental studies despite all the conceptual and logistical problems of doing so [4,151,152]. For example, given that experiments on deep sea life-forms are virtually impossible, well-designed correlational studies are needed, perhaps using new sophisticated statistical techniques, such as machine learning, which can analyse large and complex datasets [153–155]. The data situation is somewhat better for mangroves and seagrass meadows; however, so far data collection seen from a global perspective has been haphazard convenient sampling with no global systematic sampling scheme. Even worse, very little data is available for deep sea or Arctic ecosystems. Therefore, results from close relatives which inhabit shallower or temperate regions could be used to inform risk assessments. Furthermore, in order to better establishing ecological harm, new proxies are needed that can be used to assess a species’ fitness or health beyond the mere ingestion of (micro-)plastic.

### Investigating multiple stressors

The ecological harm inflicted on marine populations, species, and ecosystems is not due to plastic pollution alone, but due to multiple stressors [6]. In the real world, plastic pollution does not happen in isolation, and future research should investigate multiple impacts and stressors [151,156], possibly in interdisciplinary research teams and projects.

Mangroves are a good example. In July 2019, an oil spill in a West Javan wildlife sanctuary covered about two-thirds of all mangrove trees with oil [157]. Mangrove forests can take ten years to recover from the negative effects of oil spills or even completely vanish [158]. In addition, the same sanctuary has been polluted by high macrodebris densities (1603 items/100 m^2^ [159]). Given that many Javan mangroves also face habitat conversion, land subsidence, sediment deposition and erosion, it is obvious that plastic pollution is not the only or the main stressor in most areas, but it ‘can be the main threat to mangroves where plastic pollution is high' (C. van Bijsterveldt, in litt. 2022). Sediment deposition can actually fixate the plastic layers in place which then suffocate the roots and pneumatophores; thus, sediment deposition and plastic pollution can also work in concert (C. van Bijsterveldt, in litt. 2022).

A similar case are the mangroves of Manila Bay: historically, habitat conversion reduced their area from 54 000 hectares to 800 hectares by 1995, but recent ‘choking’ levels of plastic pollution could administer the coup de grâce to what little remains [49].

Surely, similar storylines of multiple stressors combining to damage or destroy marine ecosystems are being repeated around the world. However, we clearly do not know enough. In Indonesia alone, the vast majority of mangrove forests have not been examined for plastic pollution, let alone for the impacts of multiple stressors (C. van Bijsterveldt, in litt. 2022). Therefore, there is an urgent research need for more field work and systematic and unbiased monitoring on a global scale for all marine ecosystems, and especially the threatened ones [10,26,121], and to include multiple stressors into monitoring, correlational and experimental studies [18,160].

For example, multiple-stressor experiments were already conducted for seagrasses [67,70], for Antarctic krill [161], and for various coral reef lifeforms (bleaching x plastic pollution [162–165] or habitat degradation x plastic pollution [166]). Since many of these studies were conducted in the laboratory, field studies (e.g. [167]) should be attempted whenever possible [10]. The emerging concept of the ‘safe operating space' for ecosystems which face multiple stressors could also feasibly incorporate plastic pollution as one of the stressors included [168].

### Negative effects on human well-being

While much more research is clearly needed, it should also be self-evident that further research should not be an excuse for not taking action against exponentially increasing levels of plastic pollution [6]. Besides documenting the extent of the problem, scientific research can further spur action by actually documenting how people are affected by plastic pollution, through economic losses [169–171], through impaired health [172–174], and through loss of psychological well-being [175,176].

Action to reduce plastic pollution will only happen when large sections of the human population become sufficiently concerned about the problem [177,178]. For instance, Taiwan recently announced a widespread ban of single-use plastics due to public pressure and citizen and NGO actions [179]. To support such public actions, research which documents the impairment and loss of functions and services of marine ecosystems and how they impact human well-being should be very helpful [175,180,181]. For example, tourism is clearly negatively affected by plastic pollution [171,182,183]; one reason is the damage to the psychological benefits of clean coastlines [176], and another reason is the actual cost of cleaning beaches [184]. Such studies as these are still few and far between, even though they promise both scientific as well as socio-environmental progress.

### The need to prioritise

We again emphasise that the many research needs concerning plastic pollution should not distract from the need for environmental action. It is becoming more obvious by the day that especially coral reefs, mangroves, and Arctic ecosystems, but probably also other marine ecosystems, are all declining rapidly and will not survive the human-caused multiple stressor assault for more than a few decades [185,186]. Therefore, researchers should ask strategic questions about what research questions have the highest priority given how little time we have to save the very ecosystems which we study. For example, is it justified to study every possible combination of multiple stressors in long-term and costly mesocosm experiments?

One possibility could be to reduce the number of possible combinations of stressors to only a limited subset: e.g. no stressors, and all stressors combined. Given that currently, all stressors continue to increase (e.g. global heating, habitat fragmentation and loss, overfishing, plastic pollution, etc.), it could be argued that we might as well study the result of *all* the stressors combined, without picking apart what the contribution of each stressor is — the reason being that there is currently no indication that any of the stressors will suddenly disappear. Rather, for the foreseeable future, they will *all* continue to impact marine ecosystems. Therefore, we might as well study the endpoint of the worst-case scenario. To try to disentangle all the different possible trajectories and outcomes would be a costly and time-consuming strategy, which may not be justified given the reality on the ground. We admit that this is a contentious opinion, but it may stimulate a much-needed discussion about how to prioritise precious research resources.

## Conclusion

We should keep in mind that plastic pollution is just another manifestation of a destructive economic system and just adds another impact to the already existing cocktail of man-made impacts. Therefore, in our opinion, we need to urgently move on from the current (1) laboratory/mesocosm-based, (2) single-species focus, (3) only plastic pollution impact studies to (1) field-based, (2) population- and ecosystem-focus, (3) multiple-stressor impact studies as such studies should give us the best insights into what the future might hold (see, e.g. Rowlands et al. [151] for a good discussion of this topic). Such studies might help to steer society away from our current destructive economic system and towards a more sustainable and restorative socio-economic system [187–191].

## Summary

- Plastic pollution is a worldwide phenomenon harming all marine ecosystems
- However, the impact on some understudied marine ecosystems is less well-known
- We review the impacts of plastic pollution on mangroves, seagrass meadows, the Arctic Ocean and the deep seafloor
- Plastic pollution is an additional stressor to these already threatened ecosystems
- We give some research recommendations for the future
