# Supplementary material for: Plastic pollution of four understudied marine ecosystems: a review of mangroves, seagrass meadows, the Arctic Ocean and the deep seafloor
Source: Emerg Top Life Sci. 2022 Oct 10;6(4):371–87. doi: 10.1042/ETLS20220017 (PMC9788372; doi:10.1042/ETLS20220017)
Supplement: Supplementary Material [file ETLS-6-371-s1.pdf]

Supplement to:

Plastic pollution of four understudied marine ecosystems: a review of mangroves, seagrass meadows, the Arctic Ocean and the deep seafloor

Bruno Andreas Walther[1], Melanie Bergmann[1]

[1]Alfred Wegener Institute Helmholtz Centre for Polar and Marine Research, Bremerhaven, Germany

Text S1. Methods used for this review

We used three sources of information.

1. The online portal LITTERBASE (http://litterbase.org) is a regularly updated database on the global distribution and composition of litter pollution and its impacts on biota [1, 2]. The data held in the portal are taken from peer-reviewed publications (2459 studies in January 2021) and are fed manually into a database using standardised protocols. Striving for a comprehensive picture, the scientific literature is continuously screened for new articles, and bulk updates are performed periodically.
2. This database was then extensively analysed for the report "Impacts of plastic pollution in the oceans on marine species, biodiversity and ecosystems" [3]. For the report, further extensive literature searches were conducted using appropriate keywords.
3. To augment these two extensive libraries of publications, we conducted literature searches on Google Scholar in March 2022 using appropriate keywords such as 'plastic pollution, 'litter' in various combinations with 'ecosystem', 'ecosystem functioning', 'ecosystem services', and 'multiple stressor'. Importantly, whenever a relevant publication was found, we also checked the references cited within that publication as well as publications which cited that publication.

Many previous reviews (see Results) have already published good summaries of (1) macroplastic and microplastic abundances, compositions, and distributions, (2) possible origin and pathways, and (3) presence of plastics in biota. Therefore, we instead focused on extracting and summarizing all the published observational, correlational, or (at best) experimental evidence that plastic pollution harms these already threatened ecosystems. However, in the supplements, we also review much of the literature which merely reports on the presence of microplastics in mangroves (Text S3) and seagrasses (Text S4).

Text S2. Brief description of marine ecosystems, their ecosystem services and man-made threats

Mangroves

Mangrove forests consist of specialized salt-tolerant shrubs and small trees which can survive in brackish or saline water and are found in the transition area between the terrestrial coastlines and the ocean of the tropics and the subtropics. Estimates for the total area covered by mangrove forests in 2000 vary substantially from 83495 to 173067 km^2, with mangroves found 118 countries on all continents except Antarctica [4, 5]. An estimate for 2010 is 137600 km^2 [6], and two estimates for 2016 are 135870 km^2 [7] and 160143 km^2 [8].

Ecosystem services include coastal protection (e.g., against storm surges and tsunamis), erosion protection, food and wood production, carbon sequestration, nutrient cycling, water purification, and recreation [9-23].

Mangrove ecosystems face a combination of several stressors besides plastic pollution [24]. Habitat loss and fragmentation are probably the most important ones as up to 35% of all mangroves have already been destroyed, and possibly more, often for aquaculture or development [4, 7, 8, 18, 25-27]. However, rates of annual loss were estimated to be only 0.13% for the period of 2000-2016 which is substantially lower than estimates for pre-2000 rates [8]. Climate change and the associated sea-level rise and increased storm frequency and severity are additional and increasing stressors, leading to erosion and land subsidence [12, 18, 28-30]. Further stressors are altered hydrological regimes, eutrophication, pollution, exotic species, and overharvesting of wood [18, 31]. To maintain mangrove ecosystems will also require drastic changes in human activities and widespread conservation measures [4, 24, 32].

Sea grasses

Seagrasses are flowering marine plants which form extensive underwater meadows in shallow waters (typically down to 60 m) and constitute a unique, productive, and highly diverse ecosystem found on all continents except Antarctica [33]. They are a critical habitat for endangered dugongs, manatees, and sea turtles [34] and provide various ecosystem services: disease control, fertilizer and food production, carbon sequestration, coastal

protection, nutrient cycling, sediment production, water purification, and recreation [16, 17, 35-38]. Together with coral reefs and mangroves, seagrass meadows are one of the most productive coastal habitats [39] but their ecosystem services remain understudied in comparison to those of coral reefs and mangroves [11].

Seagrass ecosystems also face a combination of several stressors [40-42] besides plastic pollution: worsening water quality due to runoff of sediments, nutrients, changing salinity, and toxic chemicals [34]; climate change [34, 43, 44]; invasive species, disease, and physical disturbance due to dredging and boating effects [40]; and accelerating habitat loss (30-50%, see [45-48]) due to coastal development, damming, and other factors. To maintain seagrass ecosystems will also require drastic changes in human activities and widespread conservation measures [34, 49-52].

Text S3. Additional information about microplastics in mangroves

A study in Colombia compared microplastic densities in mangrove forest sediments close to and far away from populated centers [53]. 734 microplastics/1 kg of dry weight were found in the three sites near centers, and 1090 microplastics/1 kg of dry weight in the three sites far away (across all sites, the mean was 912, and the range was 31-2863).

Along the Atrato River in Colombia, microplastic density was 91.3 items/100 m^3 in four different habitats of which one was mangroves [54]. Very high microplastic concentrations were also found in six mangrove forests in Brazil, with a mean of 10782 microplastics/kg [55].

Examining the sediments of five mangrove sites in Iran, a mean density of 27.2 microplastics/1 kg of dry sediment was detected (range 19.5-34.5), with fibers and fragments being the most common types while films and pellets were much more uncommon [56].

Examining sediments sampled within seven intertidal mangroves habitats in Singapore, microplastics were found in all of them [57]. A mean of 36.7 microplastics/1 kg of dry sediment was found (range 12.0-62.8).

In Indonesian mangrove, microplastics were found up to a depth of 30 cm, with films and fibers being most abundant [58].

In a Brazilian estuary which includes mangrove areas, 26.1 microplastics/100 m^3 were detected in water samples [59]. Sampling 12 mangrove creeks in the same estuary, the density of macro- and microplastics was 4.77 items/100 m^3 [60].

In the same estuary, three catfish species had ingested plastics (18% of *Cathorops spixii* individuals, 33% of *C. agassizii* individuals, and 17% of *Sciades herzbergii* individuals) [61]. Contaminated fish contained 1-10 microplastics, and one *C. agassizii* individual was found entangled in a nylon monofilament net in 2006. In the same estuary, 64% of all individuals of an economically important fish species, the Acoupa weakfish (*Cynoscion acoupa*), had ingested microplastics, with 97% being plastic fibers [62]. In fact, all of the sampled adults were contaminated.

Thirty out of 32 species of mangrove fish species from southern China contained microplastics (mean 2.83 ± 1.84 items/individual, range 0.6-8.0 items/individual), with larger fish containing more microplastics [63]. Relatively few microplastics were also found

in mudskipper fish in mangrove forests in southern Iran [64]. In Hong Kong, every one of 49 individuals representing four crab species had microplastics in their digestive or respiratory systems, with a mean of 61 microplastics per individual [65]. Microplastics were also detected in hard clams taken from a Malaysian mangrove forest [66]. Near Jakarta, microplastics were found in five species of fish consumed by the mangrove-dwelling little black cormorant (*Phalacrocorax sulcirostris*) [67].

Text S4. Additional information about microplastics and leached chemicals in seagrass meadows

Several studies have demonstrated that seagrass meadows can trap microplastics and may thus be another sink for microplastics. These studies mostly relied on showing that there were more microplastics in the seagrass meadows than in adjacent unvegetated habitats. For example, in seagrass (*Enhalus acodoides*) meadows in Hainan, China, microplastic density ranged from 80.0 to 884.5 items/kg of dry sediment [68]. The trapping effect was also demonstrated: sediments with seagrass growing on them had two to three times more microplastics than sediments from unvegetated plots. In a later study in different localities, eight comparisons of seagrass sediments with sediments from adjacent unvegetated sites also demonstrated higher microplastic concentrations in the seagrass sediments in each case (see [69]; one-sample sign test, $P = 0.008$).

In an eelgrass bed in Orkney, Scotland, microplastics were found in samples of seawater, sediments and its associated biota, and eelgrass blades and its associated biota, with 94% of samples containing microplastics (of which > 50% were fibers) [70]. This study also showed that seagrass sediments (113000 microplastics/m^3) trapped more microplastics than clean sandy sediments (68000 microplastics/m^3).

In the Florida Keys, seven comparisons of seagrass sediments with sediments from adjacent unvegetated sites showed that, on average, seagrass sites had higher microplastic concentrations; however, the statistical difference was weak [71].

In Mediterranean seagrass meadows, microplastics accumulated mainly along the inner margin of the meadows. They were predominantly trapped there because the reduction of the current's flow velocity within the meadow facilitates sedimentation and hinders the resuspension of particles [72].

The trapping effect was further corroborated by three experimental studies. Two decades ago, the ability of seagrass meadows to trap sestonic particles was demonstrated experimentally with suspended fluorescent tracer particles (using both biological and intert tracer particles). Seagrass canopies trapped up to four times more particles than the unvegetated and plankton controls [73]. Another experimental study used eelgrass (*Zostera marina*), a common seagrass species, kept in laboratory tanks [74]. While bare sand did not retain microplastics, the seagrass canopy retained them. The probability of

retention increased with the plastic density and the canopy density and decreased with the water velocity. A similar experiment reached similar conclusions [75]. Within an experimental field flume setup, microplastics were trapped in three biogenic habitats: seagrasses, macroalgae, and scleractinian corals. More than 90% of the microplastics were trapped within the sediment which is thus the main sink for microplastics. Seagrasses had the lowest trapping potential, and corals the highest one. This trapping effect is likely due to the near-bed turbulent kinetic energy, which is a hydrodynamic process that causes sediment trapping. Less than 10% of the microplastics adhered to the benthic structures, and architectural complexity and species cuticle characteristics of these benthic structures were identified as key contributors to higher plastic adhesion.

Only two studies had less convincing or contradictory evidence concerning this trapping potential of seagrass meadows. A study in Portugal also showed that microplastics were trapped in seagrass habitats [76]. While there were more microplastics in the sediments of seagrasses than in the sediments of adjacent unvegetated plots when subtidal plots were compared, the results were reversed when intertidal plots were compared.

Mirroring the study by Huang et al. [69], eight comparisons of seagrass sediments with sediments from adjacent unvegetated sites did not show any significant differences in microplastic concentrations [77]. Despite most studies (5.5 out of 7 or 79%) showing more microplastics in seagrass sediments compared to adjacent unvegetated sediments, Unsworth et al. [77] claimed that “the contamination reflects a general build-up of microplastics in the wider environment rather than becoming concentrated within seagrass as an enhanced sink” and that “there is limited field evidence that seagrass meadows do accumulate microplastics at higher concentrations than surrounding sediments.” In our opinion, it appears to be rather subjective what should be considered limited versus considerable evidence. Fact is that the majority of studies so far contradict Unsworth et al.’s [77] conclusion. Therefore, we conclude that the current evidence points more strongly towards seagrass meadows, just like other structurally complex habitats (e.g., coral reefs and mangroves), trap microplastics and thus can be considered a sink for microplastics. Furthermore, our conclusion is backed up by experimental evidence which clearly demonstrated the trapping potential of benthic organisms with a complex architecture and rough surface [73-75].

Accordingly, numerous other studies have also detected microplastics in seagrass meadows. Examining the sediments of seagrass ecosystems in the Saudi Arabian Red Sea, great variation was detected, ranging from 10 to 160 microplastics (size of > 1 mm) per m^2 of sediment [78]. Microplastics were also found in Balearic sediments adjacent to *Posidonia oceanica* seagrass meadows [79] and in Indian sediments within seagrass meadows [80].

Microplastics were also found in the sediment of seagrass meadows along the Spanish coast (68-3819 items/kg) [81]. Interestingly, both the spatial and temporal distribution could be explained by the recent intensification of the agricultural industry in the region. Spatially, areas close to agricultural areas with more plastic-covered greenhouses had higher microplastic concentrations, and these concentrations began to rise in the 1970s when this intensive agriculture took off.

Microplastics were found in samples of sediments and benthos collected in several seagrass ecosystems in South Sulawesi, Indonesia [82]. 10 out of 51 individuals belonging to five benthos species contained microplastics, with a mean of 0.37 microplastics per individual. Microplastics were also detected in samples of seagrass blades, seawater, sediments, benthos, fishes, and invertebrates (sea cumumber, sea hare, sea urchin) collected in seagrass ecosystems in South Sulawesi, Indonesia [83-87]. 13 out of 46 individuals belonging to four fish species contained microplastics, with a mean of 0.46 microplastics per individual, and 11 out of 42 individuals belonging to three benthos species contained microplastics, with a mean of 0.38 microplastics per individual [86].

Many tropical seagrass communities are dominated by the turtle grass (*Thalassia testudinum*). In such a community near an urban center of Belize, microplastics were found on 75% of all grass blades, with 81% of microplastics being fibers [88]. Microplastics were also detected on 55-63% of the surfaces of three species of intertidal seagrasses (*Cymodocea rotundata*, *Cymodocea serrulata*, *Thalassia hemprichii*) growing around Singapore, with a little less than one microplastic item per seagrass blade [89].

Near Corsica, researchers sampled the invertebrate community colonizing a seagrass species (*Posidonia oceanica*) [90]. The invertebrates themselves are prey for fish and other species, and 27% of the invertebrates contained fibers of viscose (also called rayon, which is a cellulose-based fiber often used in clothing), with a mean of approximately one fiber per individual. Some of the dyes used to colour these fibers may be carcinogenic for vertebrates. Since microplastics are found on the vegetation and within the invertebrates

living on it, any herbivores or predators eating these food items likely also ingest microplastics. Thus microplastics are introduced into marine food webs. Corroborating this assumption was a study which found a mean of one microplastic per individual fish in the stomachs of seagrass fishes caught along Saudi Arabia's Red Sea coast [91].

A few studies also focused on chemicals leached from plastics. Examining the sediments of seagrass ecosystems in the Saudi Arabian Red Sea, various metals and PAHs were detected, but concentrations of both contaminant groups were relatively low [78]. Negative effects of BPA on the growth of a seagrass species (*Cymodocea nodosa*) were found in laboratory studies [92-94], but whether BPA has the same effect in natural ecosystems remains, to our knowledge, unknown.

References

1 Bergmann, M., Tekman, M.B., Walter, A. and Gutow, L. (2018) Tackling marine litter—LITTERBASE. In *Building bridges at the science-stakeholder interface: Towards knowledge exchange in Earth System Science* (Krause, G., ed), pp. 85-92, Springer

2 Bergmann, M., Tekman, M.B. and Gutow, L. (2017) Sea change for plastic pollution. *Nature* **544** https://doi.org/10.1038/544297a

3 Tekman, M.B., Walther, B.A., Peter, C., Gutow, L. and Bergmann, M. (2022). Impacts of plastic pollution in the oceans on marine species, biodiversity and ecosystems. WWF Germany, Berlin, Germany www.wwf.de/plastic-biodiversity-report

4 Friess, D.A., Rogers, K., Lovelock, C.E., Krauss, K.W., Hamilton, S.E., Lee, S.Y. et al. (2019) The state of the world's mangrove forests: past, present, and future. *Annu. Rev. Environ. Resour.* **44** https://doi.org/10.1146/annurev-environ-101718-033302

5 Spalding, M., Kainuma, M. and Collins, L. (2010) World atlas of mangroves. Earthscan Publications

6 Bunting, P., Rosenqvist, A., Lucas, R., Rebelo, L., Hillarides, L. and al., e. (2018) The Global Mangrove Watch—a new 2010 global baseline of mangrove extent. *Remote Sens.* **10** https://doi.org/10.3390/rs10101669

7 Worthington, T.A., Zu Ermgassen, P.S., Friess, D.A., Krauss, K.W., Lovelock, C.E., Thorley, J. et al. (2020) A global biophysical typology of mangroves and its relevance for ecosystem structure and deforestation. *Sci. Rep.* **10** https://doi.org/10.1038/s41598-020-71194-5

8 Goldberg, L., Lagomasino, D., Thomas, N. and Fatoyinbo, T. (2020) Global declines in human-driven mangrove loss. *Glob. Change Biol.* **26** https://doi.org/10.1111/gcb.15275

9 Guannel, G., Arkema, K., Ruggiero, P. and Verutes, G. (2016) The power of three: coral reefs, seagrasses and mangroves protect coastal regions and increase their resilience. *PLoS ONE* **11** https://doi.org/10.1371/journal.pone.0158094

10 Menéndez, P., Losada, I.J., Torres-Ortega, S., Narayan, S. and Beck, M.W. (2020) The global flood protection benefits of mangroves. *Sci. Rep.* **10** https://doi.org/10.1038/s41598-020-61136-6

11 Mehvar, S., Filatova, T., Dastgheib, A., De Ruyter van Steveninck, E. and Ranasinghe, R. (2018) Quantifying economic value of coastal ecosystem services: a review. *J. Mar. Sci. Eng.* **6** https://doi.org/10.3390/jmse6010005

12 Wang, Y.-S. and Gu, J.-D. (2021) Ecological responses, adaptation and mechanisms of mangrove wetland ecosystem to global climate change and anthropogenic activities. *Int. Biodeterior. Biodegradation* **162** https://doi.org/10.1016/j.ibiod.2021.105248

13 Friess, D.A., Yando, E.S., Alemu, J.B., Wong, L.-W., Soto, S.D. and Bhatia, N. (2020) Ecosystem services and disservices of mangrove forests and salt marshes. *Oceanogr. Mar. Biol.* **58** https://library.oapen.org/handle/20.500.12657/43146

14 Dahdouh-Guebas, F., Jayatissa, L.P., Di Nitto, D., Bosire, J., Lo Seen, D. and Koedam, N. (2005) How effective were mangroves as a defence against the recent tsunami? *Current Biol.* **15** https://doi.org/10.1016/j.cub.2005.06.008

15 Brander, L.M., Wagtendonk, A.J., Hussain, S.S., McVittie, A., Verburg, P.H., de Groot, R.S. et al. (2012) Ecosystem service values for mangroves in Southeast Asia: A meta-analysis and value transfer application. *Ecosyst. Serv.* **1** https://doi.org/10.1016/j.ecoser.2012.06.003

16 Himes-Cornell, A., Pendleton, L. and Atiyah, P. (2018) Valuing ecosystem services from blue forests: a systematic review of the valuation of salt marshes, sea grass beds and mangrove forests. *Ecosyst. Serv.* **30** https://doi.org/10.1016/j.ecoser.2018.01.006

17 Barbier, E.B., Hacker, S.D., Kennedy, C., Koch, E.W., Stier, A.C. and Silliman, B.R. (2011) The value of estuarine and coastal ecosystem services. *Ecol. Monogr.* **81** https://doi.org/10.1890/10-1510.1

18 Alongi, D.M. (2002) Present state and future of the world's mangrove forests. *Environ. Conserv.* **29** https://doi.org/10.1017/S0376892902000231

19 Alongi, D.M. (2012) Carbon sequestration in mangrove forests. *Carbon Manage.* **3** https://doi.org/10.4155/cmt.12.20

20 Sievers, M., Brown, C.J., Tulloch, V.J., Pearson, R.M., Haig, J.A., Turschwell, M.P. et al. (2019) The role of vegetated coastal wetlands for marine megafauna conservation. *Trends Ecol. Evol.* **34** https://doi.org/10.1016/j.tree.2019.04.004

21 Hochard, J.P., Hamilton, S. and Barbier, E.B. (2019) Mangroves shelter coastal economic activity from cyclones. *Proc. Natl. Acad. Sci. USA* **116** https://doi.org/10.1073/pnas.1820067116

22 Hamilton, S.E. and Friess, D.A. (2018) Global carbon stocks and potential emissions due to mangrove deforestation from 2000 to 2012. *Nat. Clim. Change.* **8** https://doi.org/10.1038/s41558-018-0090-4

23 Danielsen, F., Sørensen, M.K., Olwig, M.F., Selvam, V., Parish, F., Burgess, N.D. et al. (2005) The Asian tsunami: A protective role for coastal vegetation. *Science* **310** 10.1126/science.1118387

24 Turschwell, M.P., Tulloch, V.J., Sievers, M., Pearson, R.M., Andradi-Brown, D.A., Ahmadia, G.N. et al. (2020) Multi-scale estimation of the effects of pressures and drivers on mangrove forest loss globally. *Biol. Converv.* **247** https://doi.org/10.1016/j.biocon.2020.108637

25 Bryan-Brown, D.N., Connolly, R.M., Richards, D.R., Adame, F., Friess, D.A. and Brown, C.J. (2020) Global trends in mangrove forest fragmentation. *Sci. Rep.* **10** https://doi.org/10.1038/s41598-020-63880-1

26 Valiela, I., Bowen, J.L. and York, J.K. (2001) Mangrove forests: One of the world's threatened major tropical environments. *BioScience* **51** https://doi.org/10.1641/0006-3568(2001)051[0807:MFOOTW]2.0.CO;2

27 Millennium Ecosystem Assessment (2005) Ecosystems and human well-being: A framework for assessment. Island Press, Washington, D.C., USA

28 Sippo, J.Z., Lovelock, C.E., Santos, I.R., Sanders, C.J. and Maher, D.T. (2018) Mangrove mortality in a changing climate: An overview. *Estuar. Coast. Shelf Sci.* **215** https://doi.org/10.1016/j.ecss.2018.10.011

29 Gilman, E.L., Ellison, J., Duke, N.C. and Field, C. (2008) Threats to mangroves from climate change and adaptation options: a review. *Aquat. Bot.* **89** https://doi.org/10.1016/j.aquabot.2007.12.009

30 Lovelock, C.E., Cahoon, D.R., Friess, D.A., Guntenspergen, G.R., Krauss, K.W., Reef, R. et al. (2015) The vulnerability of Indo-Pacific mangrove forests to sea-level rise. *Nature* **526** https://doi.org/10.1038/nature15538

31 Biswas, S.R., Biswas, P.L., Limon, S.H., Yan, E.-R., Xu, M.-S. and Khan, M.S.I. (2018) Plant invasion in mangrove forests worldwide. *Forest Ecol. Manage.* **429** https://doi.org/10.1016/j.foreco.2018.07.046

32 Romañach, S.S., DeAngelis, D.L., Koh, H.L., Li, Y., Teh, S.Y., Barizan, R.S.R. et al. (2018) Conservation and restoration of mangroves: Global status, perspectives, and prognosis. *Ocean Coast. Manage.* **154** https://doi.org/10.1016/j.ocecoaman.2018.01.009

33 Boström, C., Jackson, E.L. and Simenstad, C.A. (2006) Seagrass landscapes and their effects on associated fauna: a review. *Estuar. Coast. Shelf Sci.* **68** https://doi.org/10.1016/j.ecss.2006.01.026

34 Orth, R.J., Carruthers, T.J., Dennison, W.C., Duarte, C.M., Fourqurean, J.W., Heck, K.L. et al. (2006) A global crisis for seagrass ecosystems. *BioScience* **56** https://doi.org/10.1641/0006-3568(2006)56[987:AGCFSE]2.0.CO;2

35 Ruiz-Frau, A., Gelcich, S., Hendriks, I., Duarte, C.M. and Marbà, N. (2017) Current state of seagrass ecosystem services: research and policy integration. *Ocean Coast. Manage.* **149** https://doi.org/10.1016/j.ocecoaman.2017.10.004

36 Ondiviela, B., Losada, I.J., Lara, J.L., Maza, M., Galván, C., Bouma, T.J. et al. (2014) The role of seagrasses in coastal protection in a changing climate. *Coast. Eng.* **87** https://doi.org/10.1016/j.coastaleng.2013.11.005

37 Luisetti, T., Jackson, E.L. and Turner, R.K. (2013) Valuing the European 'coastal blue carbon' storage benefit. *Mar. Pollut. Bull.* **71** https://doi.org/10.1016/j.marpolbul.2013.03.029

38 Mtwana Nordlund, L., Koch, E.W., Barbier, E.B. and Creed, J.C. (2016) Seagrass ecosystem services and their variability across genera and geographical regions. *PLoS ONE* **11** https://doi.org/10.1371/journal.pone.0169942

39 Short, F.T. and Wylie-Echeverria, S. (1996) Natural and human-induced disturbance of seagrasses. *Environ. Conserv.* **23** https://doi.org/10.1017/S0376892900038212

40 Unsworth, R.K., Collier, C.J., Waycott, M., Mckenzie, L.J. and Cullen-Unsworth, L.C. (2015) A framework for the resilience of seagrass ecosystems. *Mar. Pollut. Bull.* **100** https://doi.org/10.1016/j.marpolbul.2015.08.016

41 Griffiths, L.L., Connolly, R.M. and Brown, C.J. (2020) Critical gaps in seagrass protection reveal the need to address multiple pressures and cumulative impacts. *Ocean Coast. Manage.* **183** https://doi.org/10.1016/j.ocecoaman.2019.104946

42 Ceccherelli, G., Oliva, S., Pinna, S., Piazzi, L., Procaccini, G., Marin-Guirao, L. et al. (2018) Seagrass collapse due to synergistic stressors is not anticipated by phenological changes. *Oecologia* **186** https://doi.org/10.1007/s00442-018-4075-9

43 Jordà, G., Marbà, N. and Duarte, C.M. (2012) Mediterranean seagrass vulnerable to regional climate warming. *Nat. Clim. Change* **2** https://doi.org/10.1038/nclimate1533

44 Nowicki, R.J., Thomson, J.A., Burkholder, D.A., Fourqurean, J.W. and Heithaus, M.R. (2017) Predicting seagrass recovery times and their implications following an extreme climate event. *Mar. Ecol. Prog. Ser.* **567** https://doi.org/10.3354/meps12029

45 Macusi, E.D., Deepananda, A.K., Conte, A.R., Katikiro, R.E., Fadli, N. and Jimenez, L.A. (2011) Human induced degradation of coastal resources in Asia Pacific and implications on management and food security. *J. Nat. Stud.* **9** https://d1wqtxts1xzle7.cloudfront.net/45883661/HUMAN_INDUCED_DEGRADATION_OF_COASTAL_RES20160523-16683-b1teg6-with-cover-page-v2.pdf?Expires=1662325457&Signature=Yf6DKgsJAz9yUwTx2y9YXIXe9fpmrB~Av4vJPR6WM1QTyBDRTs1ymG-IDOm6Zu80wHXeH9IuhSvicmuBcPJXNLuf5i0z1TgXN4MpSrO9VV680j7h3-SwTO9zbsP4CTGGbKFZ8sGEVZBaley~CwHdcrynkPrld-nn3nzEhSjvGjUPr08K5qyTqqPeuBzmCF~~IBYk6rqmYeghfz7MDCeIANot34H6aEYgrxzjeTOtQUrxV5JW9Rw7GbK~R2LqQKbCKwGkGnaYYodPv4xb8LyjfvR5qujvRc-VGmz-U2N1gfukdZePrenRbCia21QlwI~2eA23iaVU2bOkcBSKct5Pkw__&Key-Pair-Id=APKAJLOHF5GGSLRBV4ZA

46 Short, F.T. and Wyllie-Echeverria, S. (2000) Global seagrass declines and effects of climate change. In *Seas at the Millennium: An environmental evaluation, vol. 3* (Sheppard, C., ed), pp. 10-11, Elsevier Science, Amsterdam, Netherlands

47 Waycott, M., Duarte, C.M., Carruthers, T.J., Orth, R.J., Dennison, W.C., Olyarnik, S. et al. (2009) Accelerating loss of seagrasses across the globe threatens coastal ecosystems. *Proc. Natl. Acad. Sci. USA* **106** https://doi.org/10.1073/pnas.0905620106

48 Jackson, J.B.C. (2008) Ecological extinction and evolution in the brave new ocean. *Proc. Natl. Acad. Sci. USA* **105** https://doi.org/10.1073/pnas.0802812105

49 Bonanno, G. and Orlando-Bonaca, M. (2020) Marine plastics: What risks and policies exist for seagrass ecosystems in the Plasticene? *Mar. Pollut. Bull.* **158** https://doi.org/10.1016/j.marpolbul.2020.111425

50 Cullen-Unsworth, L.C. and Unsworth, R.K. (2018) A call for seagrass protection. *Science* **361** 10.1126/science.aat7318

51 Tan, Y.M., Dalby, O., Kendrick, G.A., Statton, J., Sinclair, E.A., Fraser, M.W. et al. (2020) Seagrass restoration is possible: Insights and lessons from Australia and New Zealand. *Front. Mar. Sci.* **7** https://doi.org/10.3389/fmars.2020.00617

52 Unsworth, R.K., McKenzie, L.J., Collier, C.J., Cullen-Unsworth, L.C., Duarte, C.M., Eklöf, J.S. et al. (2019) Global challenges for seagrass conservation. *Ambio* **48** https://doi.org/10.1007/s13280-018-1115-y

53 Garcés-Ordóñez, O., Castillo-Olaya, V.A., Granados-Briceño, A.F., Blandón García, L.M. and Espinosa Díaz, L.F. (2019) Marine litter and microplastic pollution on mangrove soils of the Ciénaga Grande de Santa Marta, Colombian Caribbean. *Mar. Pollut. Bull.* **145** https://doi.org/10.1016/j.marpolbul.2019.06.058

54 Correa-Herrera, T., Barletta, M., Lima, A.R.A., Jiménez-Segura, L.F. and Arango-Sánchez, L.B. (2017) Spatial distribution and seasonality of ichthyoplankton and anthropogenic debris in a river delta in the Caribbean Sea. *J. Fish Biol.* **90** http://doi.org/10.1111/jfb.13243

55 da Silva Paes, E., Gloaguen, T.V., da Conceição Silva, H.d.A., Duarte, T.S., de Almeida, M.d.C., Costa, O.D.A.V. et al. (2022) Widespread microplastic pollution in mangrove soils of Todos os Santos Bay, northern Brazil. *Environ. Res.* **210** https://doi.org/10.1016/j.envres.2022.112952

56 Naji, A., Nuri, M., Amiri, P. and Niyogi, S. (2019) Small microplastic particles (S-MPPs) in sediments of mangrove ecosystem on the northern coast of the Persian Gulf. *Mar. Pollut. Bull.* **146** https://doi.org/10.1016/j.marpolbul.2019.06.033

57 Nor, M.N.H. and Obbard, J.P. (2014) Microplastics in Singapore's coastal mangrove ecosystems. *Mar. Pollut. Bull.* **79** http://dx.doi.org/10.1016/j.marpolbul.2013.11.025

58 Hastuti, A.R., Yulianda, F. and Wardianto, Y. (2014) Spatial distribution of marine debris in mangrove ecosystem of Pantai Indah Kapuk, Jakarta. *Bonorowo Wetl.* **4** 10.13057/bonorowo/w040203

59 Lima, A.R.A., Barletta, M. and Costa, M.F. (2015) Seasonal distribution and interactions between plankton and microplastics in a tropical estuary. *Estuar. Coast. Shelf Sci.* **165** http://dx.doi.org/10.1016/j.ecss.2015.05.018

60 Lima, A.R.A., Barletta, M., Costa, M.F., Ramos, J.A.A., Dantas, D.V., Melo, P.A.M.C. et al. (2015) Changes in the composition of ichthyoplankton assemblage and plastic debris in mangrove creeks relative to moon phases. *J. Fish Biol.* **89** 10.1111/jfb.12838

61 Possatto, F.E., Barletta, M., Costa, M.F., do Sul, J.A.I. and Dantas, D.V. (2011) Plastic debris ingestion by marine catfish: An unexpected fisheries impact. *Mar. Pollut. Bull.* **62** 10.1016/j.marpolbul.2011.01.036

62 Ferreira, G.V., Barletta, M., Lima, A.R., Dantas, D.V., Justino, A.K. and Costa, M.F. (2016) Plastic debris contamination in the life cycle of Acoupa weakfish (*Cynoscion acoupa*) in a tropical estuary. *ICES J. Mar. Sci.* **73** https://doi.org/10.1093/icesjms/fsw108

63 Huang, J.-S., Koongolla, J.B., Li, H.-X., Lin, L., Pan, Y.-F., Liu, S. et al. (2020) Microplastic accumulation in fish from Zhanjiang mangrove wetland, South China. *Sci. Total Environ.* **708** https://doi.org/10.1016/j.scitotenv.2019.134839

64 Maghsodian, Z., Sanati, A.M., Ramavandi, B., Ghasemi, A. and Sorial, G.A. (2020) Microplastics accumulation in sediments and *Periophthalmus waltoni* fish, mangrove forests in southern Iran. *Chemosphere* **264** https://doi.org/10.1016/j.chemosphere.2020.128543

65 Not, C., Lui, C.Y.I. and Cannicci, S. (2020) Feeding behavior is the main driver for microparticle intake in mangrove crabs. *Limnol. Oceanogr. Lett.* **5** https://doi.org/10.1002/lol2.10143

66 Hamid, F.S., Jia, W. and Zakaria, R.M. (2020) Microplastics abundance and uptake by *Meretrix lyrata* (hard clam) in mangrove forest. *JESTEC* **52** 10.5614/j.eng.technol.sci.2020.52.3.10

67 Susanti, N., Mardiastuti, A. and Hariyadi, S. (2022) Microplastics in fishes as seabird preys in Jakarta Bay Area. *IOP Conf. Ser.: Earth Environ. Sci.* **967** 10.5614/j.eng.technol.sci.2020.52.3.10

68 Huang, Y., Xiao, X., Xu, C., Perianen, Y.D., Hu, J. and Holmer, M. (2020) Seagrass beds acting as a trap of microplastics-Emerging hotspot in the coastal region? *Environ. Pollut.* **257** https://doi.org/10.1016/j.envpol.2019.113450

69 Huang, Y., Xiao, X., Effiong, K., Xu, C., Su, Z., Hu, J. et al. (2021) New insights into the microplastic enrichment in the blue carbon ecosystem: evidence from seagrass meadows and mangrove forests in coastal South China Sea. *Environ. Sci. Technol.* **55** https://doi.org/10.1021/acs.est.0c07289

70 Jones, K.L., Hartl, M.G., Bell, M.C. and Capper, A. (2020) Microplastic accumulation in a *Zostera marina* L. bed at Deerness Sound, Orkney, Scotland. *Mar. Pollut. Bull.* **152** https://doi.org/10.1016/j.marpolbul.2020.110883

71 Plee, T.A. and Pomory, C.M. (2020) Microplastics in sandy environments in the Florida Keys and the panhandle of Florida, and the ingestion by sea cucumbers (Echinodermata: Holothuroidea) and sand dollars (Echinodermata: Echinoidea). *Mar. Pollut. Bull.* **158** https://doi.org/10.1016/j.marpolbul.2020.111437

72 Navarrete-Fernández, T., Bermejo, R., Hernández, I., Deidun, A., Andreu-Cazenave, M. and Cózar, A. (2022) The role of seagrass meadows in the coastal trapping of litter. *Mar. Pollut. Bull.* **174** https://doi.org/10.1016/j.marpolbul.2021.113299

73 Agawin, N.S. and Duarte, C.M. (2002) Evidence of direct particle trapping by a tropical seagrass meadow. *Estuaries* **25** https://doi.org/10.1007/BF02692217

74 de los Santos, C.B., Krång, A.-S. and Infantes, E. (2021) Microplastic retention by marine vegetated canopies: Simulations with seagrass meadows in a hydraulic flume. *Environ. Pollut.* **269** https://doi.org/10.1016/j.envpol.2020.116050

75 de Smit, J.C., Anton, A., Martin, C., Rossbach, S., Bouma, T.J. and Duarte, C.M. (2021) Habitat-forming species trap microplastics into coastal sediment sinks. *Sci. Total Environ.* **772** https://doi.org/10.1016/j.scitotenv.2021.145520

76 Cozzolino, L., Nicastro, K.R., Zardi, G.I. and Carmen, B. (2020) Species-specific plastic accumulation in the sediment and canopy of coastal vegetated habitats. *Sci. Total. Environ.* **723** https://doi.org/10.1016/j.scitotenv.2020.138018

77 Unsworth, R.K., Higgs, A., Walter, B., Cullen-Unsworth, L.C., Inman, I. and Jones, B.L. (2021) Canopy accumulation: are seagrass meadows a sink of microplastics? *Oceans* **2** https://doi.org/10.3390/oceans2010010

78 Ruiz-Compean, P., Ellis, J., Cúrdia, J., Payumo, R., Langner, U., Jones, B. et al. (2017) Baseline evaluation of sediment contamination in the shallow coastal areas of Saudi Arabian Red Sea. *Mar. Pollut. Bull.* **123** https://doi.org/10.1016/j.marpolbul.2017.08.059

79 Alomar, C., Estarellas, F. and Deudero, S. (2016) Microplastics in the Mediterranean Sea: Deposition in coastal shallow sediments, spatial variation and preferential grain size. *Mar. Environ. Res.* **115** http://dx.doi.org/10.1016/j.marenvres.2016.01.005

80 Jeyasanta, K.I., Patterson, J., Grimsditch, G. and Edward, J.P. (2020) Occurrence and characteristics of microplastics in the coral reef, sea grass and near shore habitats of Rameswaram Island, India. *Mar. Pollut. Bull.* **160** https://doi.org/10.1016/j.marpolbul.2020.111674

81 Dahl, M., Bergman, S., Björk, M., Diaz-Almela, E., Granberg, M., Gullström, M. et al. (2021) A temporal record of microplastic pollution in Mediterranean seagrass soils. *Environ. Pollut.* **273** https://doi.org/10.1016/j.envpol.2021.116451

82 Tahir, A., Samawi, M., Sari, K., Hidayat, R., Nimzet, R., Wicaksono, E. et al. (2019) Studies on microplastic contamination in seagrass beds at Spermonde Archipelago of Makassar Strait, Indonesia. *J. Phys.: Conf. Ser.* **1341** 10.1088/1742-6596/1341/2/022008

83 Idris, F., Febrianto, T., Hidayati, J. and Nugraha, A. (2022) Microplastic abundance in sea cucumber at seagrass ecosystem of Bintan Island and surrounding area, Indonesia. *IOP Conf. Ser.: Earth Environ. Sci.* **967** 10.1088/1755-1315/967/1/012009

84 Sawalman, R., Werorilangi, S., Ukkas, M., Mashoreng, S., Yasir, I. and Tahir, A. (2021) Microplastic abundance in sea urchins (*Diadema setosum*) from seagrass beds of Barranglompo Island, Makassar, Indonesia. *IOP Conf. Ser.: Earth Environ. Sci.* **763** 10.1088/1755-1315/763/1/012057

85 Priscilla, V., Sedayu, A. and Patria, M. (2019) Microplastic abundance in the water, seagrass, and sea hare *Dolabella auricularia* in Pramuka Island, Seribu Islands, Jakarta Bay, Indonesia. *J. Physics Conf. Ser.* **1402** 10.1088/1742-6596/1402/3/033073

86 Tahir, A., Soeprapto, D., Sari, K., Wicaksono, E. and Werorilangi, S. (2020) Microplastic assessment in seagrass ecosystem at Kodingareng Lompo Island of Makassar City. *IOP Conf. Ser.: Earth Environ. Sci.* **564** 10.1088/1755-1315/564/1/012032

87 Datu, S.S., Supriadi, S. and Tahir, A. (2019) Microplastic in *Cymodocea rotundata* seagrass blades. *Int. J. Environ. Agric. Biotechnol.* **4** https://dx.doi.org/10.22161/ijeab.46.21

88 Goss, H., Jaskiel, J. and Rotjan, R. (2018) *Thalassia testudinum* as a potential vector for incorporating microplastics into benthic marine food webs. *Mar. Pollut. Bull.* **135** https://doi.org/10.1016/j.marpolbul.2018.08.024

89 Seng, N., Lai, S., Fong, J., Saleh, M.F., Cheng, C., Cheok, Z.Y. et al. (2020) Early evidence of microplastics on seagrass and macroalgae. *Mar. Freshw. Res.* **71** https://doi.org/10.1071/MF19177

90 Remy, F.o., Collard, F., Gilbert, B., Compère, P., Eppe, G. and Lepoint, G. (2015) When microplastic is not plastic: the ingestion of artificial cellulose fibers by macrofauna living in seagrass macrophytodetritus. *Environ. Sci. Technol.* **49** https://doi.org/10.1021/acs.est.5b02005

91 Baalkhuyur, F.M., Bin Dohaish, E.J.A., Elhalwagy, M.E.A., Alikunhi, N.M., AlSuwailem, A.M., Røstad, A. et al. (2018) Microplastic in the gastrointestinal tract of fishes along the Saudi Arabian Red Sea coast. *Mar. Pollut. Bull.* **131** https://doi.org/10.1016/j.marpolbul.2018.04.040

92 Adamakis, I.-D.S., Malea, P. and Panteris, E. (2018) The effects of Bisphenol A on the seagrass *Cymodocea nodosa*: Leaf elongation impairment and cytoskeleton disturbance. *Ecotoxicol. Environ. Saf.* **157** https://doi.org/10.1016/j.ecoenv.2018.04.005

93 Malea, P., Kokkinidi, D., Kevrekidou, A. and Adamakis, I.-D.S. (2020) Environmentally relevant bisphenol A concentrations effects on the seagrass *Cymodocea nodosa* different parts elongation:

perceptive assessors of toxicity. *Environ. Sci. Pollut. Res.* **27** https://doi.org/10.1007/s11356-019-07443-6

94 Adamakis, I.-D.S., Malea, P., Sperdouli, I., Panteris, E., Kokkinidi, D. and Moustakas, M. (2021) Evaluation of the spatiotemporal effects of bisphenol A on the leaves of the seagrass Cymodocea nodosa. *J. Hazard. Mater.* **404** https://doi.org/10.1016/j.jhazmat.2020.124001
